# Classification of four distinct osteoarthritis subtypes with a knee joint tissue transcriptome atlas

**DOI:** 10.1038/s41413-020-00109-x

**Published:** 2020-11-12

**Authors:** Chunhui Yuan, Zongyou Pan, Kun Zhao, Jun Li, Zixuan Sheng, Xudong Yao, Hua Liu, Xiaolei Zhang, Yang Yang, Dongsheng Yu, Yu Zhang, Yuzi Xu, Zhi-Yong Zhang, Tianlong Huang, Wanlu Liu, Hongwei Ouyang

**Affiliations:** 1 Dr. Li Dak Sum & Yip Yio Chin Center for Stem Cells and Regenerative Medicine, and Department of Orthopedic Surgery of the Second Affiliated Hospital, Zhejiang University School of Medicine, Hangzhou, China; 2 Zhejiang University-University of Edinburgh Institute, Zhejiang University School of Medicine, and Key Laboratory of Tissue Engineering and Regenerative Medicine of Zhejiang Province, Zhejiang University School of Medicine, Hangzhou, China; 3 Department of Sports Medicine, Zhejiang University School of Medicine, Hangzhou, China; 4 Department of Orthopedics, The Second Affiliated Hospital and Yuying Children’s Hospital of Wenzhou Medical University, Wenzhou, China; 5 China Orthopedic Regenerative Medicine Group (CORMed), Hangzhou, China; 6 Department of Orthopedics, Zhejiang Provincial People’s Hospital, Hangzhou Medical College, Hangzhou, China; 7 Translational Research Centre of Regenerative Medicine and 3D Printing Technologies of Guangzhou Medical University, State Key Laboratory of Respiratory Disease, The Third Affiliated Hospital of Guangzhou Medical University, Guangzhou, China; 8 The Second Xiangya Hospital of Central South University, Changsha, China

**Keywords:** Pathogenesis, Bone quality and biomechanics, Metabolic disorders

## Abstract

The limited molecular classifications and disease signatures of osteoarthritis (OA) impede the development of prediagnosis and targeted therapeutics for OA patients. To classify and understand the subtypes of OA, we collected three types of tissue including cartilage, subchondral bone, and synovium from multiple clinical centers and constructed an extensive transcriptome atlas of OA patients. By applying unsupervised clustering analysis to the cartilage transcriptome, OA patients were classified into four subtypes with distinct molecular signatures: a glycosaminoglycan metabolic disorder subtype (C1), a collagen metabolic disorder subtype (C2), an activated sensory neuron subtype (C3), and an inflammation subtype (C4). Through ligand-receptor crosstalk analysis of the three knee tissue types, we linked molecular functions with the clinical symptoms of different OA subtypes. For example, the Gene Ontology functional term of vasculature development was enriched in the subchondral bone-cartilage crosstalk of C2 and the cartilage-subchondral bone crosstalk of C4, which might lead to severe osteophytes in C2 patients and apparent joint space narrowing in C4 patients. Based on the marker genes of the four OA subtypes identified in this study, we modeled OA subtypes with two independent published RNA-seq datasets through random forest classification. The findings of this work contradicted traditional OA diagnosis by medical imaging and revealed distinct molecular subtypes in knee OA patients, which may allow for precise diagnosis and treatment of OA.

## Introduction

Osteoarthritis (OA) is one of the most common degenerative joint diseases with increased incidence in the current aging population.^1^ Knee OA is characterized by pathological changes in most tissues of the joint, including cartilage degradation, synovial inflammation, and subchondral bone structure alteration, which ultimately lead to a narrow joint space and osteophytes, resulting in severe destruction and impaired function.^2–4^ Current treatments for OA mainly focus on symptomatic relief of pain or joint replacement surgery at the late stage. No pharmaceutical agents are capable of slowing down or halting the progression of OA.^5^ Advanced therapeutics such as autologous chondrocyte implantation (ACI) and matrix-based autologous chondrocyte implantation (MACI) produce improvements in more than 80% of OA patients. However, the efficacies of ACI and MACI in an individual patient are varied and unpredictable. The development of efficient and predictable therapeutics is limited by the limited knowledge on OA classification and the mechanisms involved.^6^ Current diagnostic and treatment strategies are still “one rule applied to all patients”,^7^ which indicates the need for research on the classification of OA subtypes. To date, clinical OA classification is based on clinical symptoms such as etiological elements, onset of position, region of influence, and other factors observed in the clinic. Unfortunately, most of these features and histopathology fail to elucidate the pathophysiological changes of OA or predict the outcome of OA treatment.^8,9^ Gene expression information together with clinical data has been useful for the classification of disease subtypes.^10,11^ Therefore, it is logical and feasible to use transcriptome data for OA subtype classification.

Cartilage with optimal extracellular matrix (ECM) function requires the maintenance of a delicate balance between anabolic and catabolic activities.^12^ Local cartilage damage is the most typical phenotype of knee OA lesions. Therefore, we investigated pathological results and defined subtypes according to the current state of OA cartilage. On the other hand, pathological results for OA cartilage and joint crosstalk reflect the accumulative effect of various factors related to both internal and external conditions. Hence, the transcriptomes of the subchondral bone tissue and synovial tissues in the joint are also essential for understanding OA subtypes. Here, we created a transcriptome atlas of articular tissues to identify OA subtypes with remarkable heterogeneity in cartilage and understand the function of cartilage, subchondral bone, and synovial crosstalk in OA symptoms.

## Results

### Identification of four OA subtypes with different metabolic activities

**Fig. 1:**
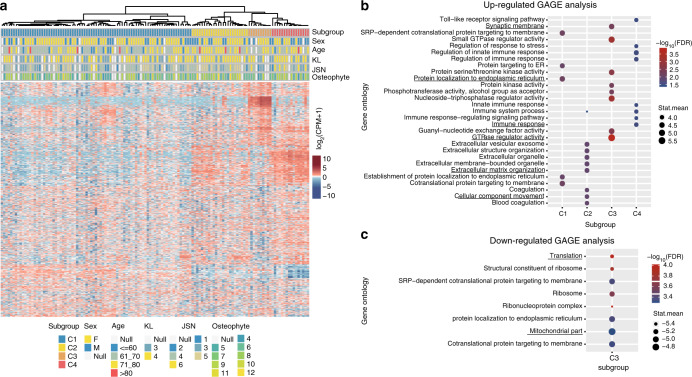
Identification of subtypes from OA cartilage RNA sequencing data. **a** Heatmap visualization of genes across 131 OA individuals revealed four distinct subtypes (C1–C4). **b** The upregulated Gene Ontology (GO) terms of each subtype were determined using GAGE. **c** The downregulated GO terms of C3 were determined using GAGE. In GAGE analysis, stat.mean represents the magnitude of GO term level changes and indicates the direction of the changes

To understand the heterogeneity of OA and identify potential subtypes, we used the next-generation sequencing technique BRB-seq^13^ to screen articular cartilage gene expression. After rigorous quality control (Supplementary Methods), 131 OA cartilage samples and four control cartilage samples were identified for further analysis. To identify OA subtypes from such a high-dimensionality dataset, we applied the unsupervised clustering method SC3^14^ combining multiple clustering solutions to identify subclusters of OA. Based on the top 4 000 most variable genes, 131 OA patients were classified into four subtypes: 81 (61.8%) into cluster 1 (C1), 24 (18.3%) into cluster 2 (C2), 10 (7.6%) into cluster 3 (C3) and 16 (12.2%) into cluster 4 (C4) (Fig. 1a). We used the GAGE method^15^ to identify the enriched functional gene set for each cluster (Supplementary Methods). The results showed that protein localization to the endoplasmic reticulum was one of the significantly upregulated Gene Ontology (GO) terms of C1, ECM organization and cellular component movement were the significantly upregulated GO terms of C2, GTPase regulator activity and synaptic membrane were the significantly upregulated GO terms of C3, and immune response was one of the significantly upregulated GO terms of C4 (Fig. 1b). C3 had significantly downregulated GO terms, such as translation and mitochondrial part (Fig. 1c), suggesting repression of metabolic processes in C3 in contrast to the active metabolism status in the other three clusters.

### Activated neuron regulation subtype

We found that various genes, such as *GRIK2*, *GRM7*, *GRID2*, and *NRXN1*, in the synapse GO term were highly expressed in C3 (Supplementary Fig. 1a). Few studies have explored scenery neuron-associated genes in OA. To determine whether neuron-related genes expressed in cartilage tissues could be found in other studies, we analyzed two publicly available gene expression omnibus (GEO) datasets containing cartilage RNA-seq data (GSE114007^16^ and GDS2809^17^) and found that many synapse assembly-related genes were expressed (Supplementary Table 1). However, genes associated with angiogenesis were not highly expressed in C3 (Supplementary Fig. 1b).

### Three activated metabolic disorder subtypes

**Fig. 2:**
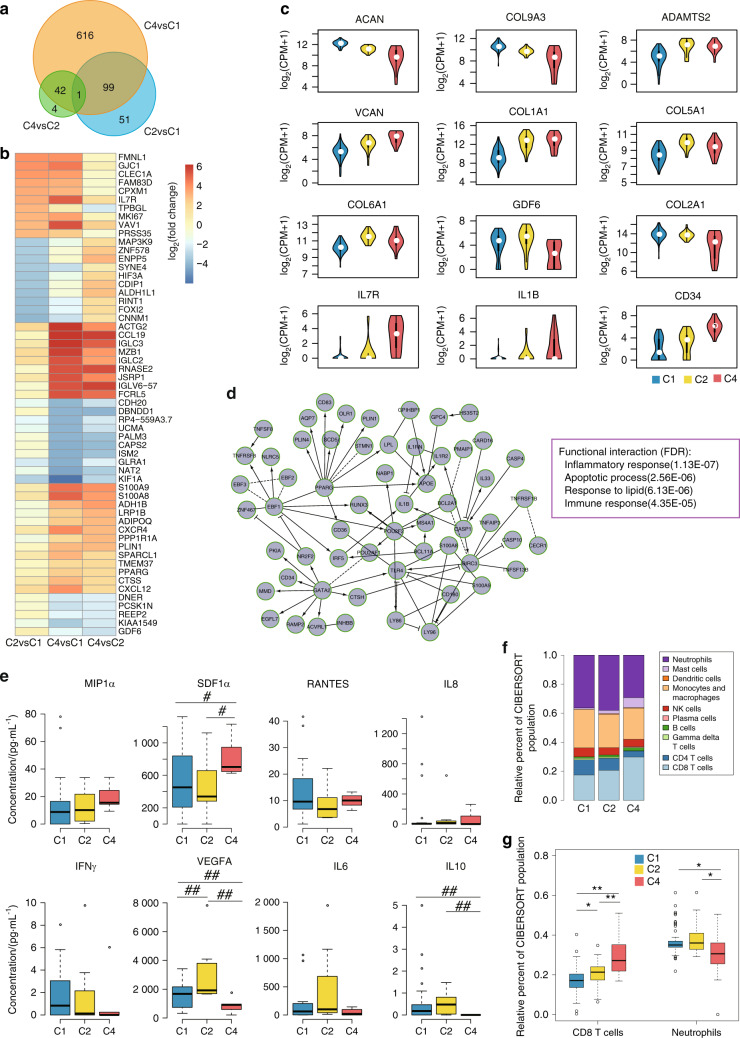
Characterization of metabolically disordered OA subtypes. **a** Venn diagram of differentially expressed protein-coding genes (DEGs). **b** Heatmap of the top 10 differentially upregulated and downregulated protein-coding genes. **c** Violin plots showing the expression of cartilage development- and OA-related genes. **d** The largest functional interaction (FI) network module of differentially expressed genes between C4 and C2. **e** Box plot of protein expression in hydrarthrosis. **f** CIBERSORT-inferred relative fractions of different immune cell types across the three metabolically disordered OA subtypes. **g** Box plot of the relative fractions of CD8+ T cells and neutrophils in OA subtypes. (Wilcoxon rank-sum test; ^#^represents FDR ≤ 0.1, ^##^represents FDR ≤ 0.05, *represents FDR ≤ 0.01, and **represents FDR ≤ 0.001)

To investigate the three metabolically disordered OA subtypes, DESeq2^18^ was applied to identify differentially expressed protein-coding genes (DEGs) among C1, C2, and C4. Overall, 151 DEGs were found between C2 and C1 (C2_vs_C1), 756 DEGs were found between C4 and C1 (C4_vs_C1), and 47 DEGs were found between C4 and C2 (C4_vs_C2), with cut-offs of a fold change greater than 4 and a false discovery rate (FDR) less than 0.05. C2_vs_C1 and C4_vs_C1 shared 100 DEGs, while 43 DEGs were common between C4_vs_C1 and C4_vs_C2, and one DEG was common between C2_vs_C1, C4_vs_C1 and C4_vs_C2 (Fig. 2a). The expression fold changes of the top 10 DEGs in each comparison were visualized (Fig. 2b). This result illustrates that C4 and C2 are the most similar.

The DEG analysis results indicated that *aggrecan* (*ACAN*) and *COL9A3* levels were upregulated in C1 compared with C2 and C4, while *ADAMTS2* and *versican* (*VCAN*) levels were downregulated in C1. *COL1A1* was similarly expressed in C1 and C2, while *COL5A1*, *COL6A1*, and *GDF6* were highly expressed in C2 (Fig. 2c). In C4, the expression of *COL2A1* was low, while the expression of *IL1β*, *IL7*, and *CD34* was high (Fig. 2c). The gene expression pattern of C4 suggested that a severe level of inflammation might be present in C4 patients. The results also showed that a large number of the DEGs were components of the ECM, which may imply the existence of distinct cartilage ECMs among the OA subtypes.

To evaluate the function of the DEGs in each cluster, we used Cytoscape to construct functional interaction (FI) networks based on the Reactome pathway database.^19^ Four FI network modules of C4_vs_C1 DEGs were identified (Supplementary Fig. 2a), and the largest FI network module represented the inflammatory response, apoptotic process, response to lipid and immune response (Fig. 2d). The other three FI network modules were enriched for the innate immune response, ECM organization and positive regulation of GTPase activity. The largest module of the FI network constructed with C2_vs_C1 DEGs was significantly enriched in ECM organization and the collagen catabolic process (Supplementary Fig. 2b). The largest module of the FI network constructed with C4_vs_C2 DEGs was enriched in the innate immune response, negative regulation of the leukocyte apoptotic process and the cellular response to a cytokine stimulus (Supplementary Fig. 2c).

To characterize the subtypes at the protein level, we analyzed secreted proteins in the synovial fluid using ELISA (Fig. 2e). MIP1α, SDF1α, RANTES, and IL8 are chemoattractants for immunocytes. IFNγ and IL6 are cytokines with essential immunoregulatory functions.^20,21^ Our ELISA results showed higher expression of IFNγ in C1, VEGFA and IL6 in C2, and MIP1α, SDF1α, and IL8 in C4 (Fig. 2e). IL10-mediated inhibition of the synthesis of a number of cytokines^22^ was significantly downregulated in C4. This result demonstrated that the three metabolically disordered OA subtypes secreted different inflammatory molecules, which might be linked to different immune responses in each subtype. Subsequently, we investigated the relative leukocyte fractions in these three subtypes using CIBERSORT^23,24^ and found a higher relative fraction of neutrophils in C1 and a higher relative fraction of CD8+ T cells in C4 (Fig. 2f, g). The results may suggest the existence of an adaptive inflammatory response in C4 OA patients (detailed statistical test results are shown in Supplementary Table 2).

### Tissue crosstalk in the whole OA knee

**Fig. 3:**
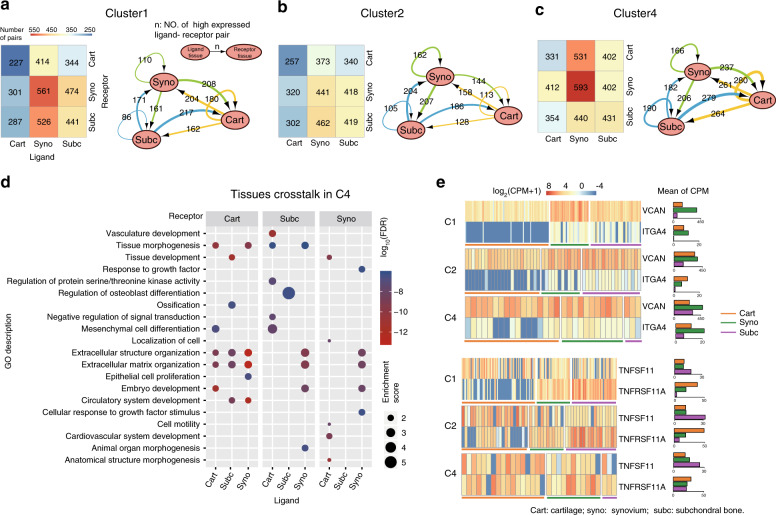
Tissue crosstalk in the OA joint. **a**–**c** Heatmap showing the number of expressed ligand-receptor pairs of all tissue crosstalk pairs in each subtype (left panel). Network visualizing the number of potential tissue-tissue crosstalk pairs in each subtype (right panel). Nodes represent tissue types, edges are proportional to the number of highly expressed ligand-receptor pairs, and arrows designate the link direction. **a** Subtype C1. **b** Subtype C2. **c** Subtype C4. **d** Enriched GO terms of all tissue crosstalk pairs in C4. **e** Heatmap (left panel) showing the gene expression of two ligand-receptor pairs, *VCAN*-*ITGA4* and *TNFSF11*-*TNFRSF11A*, in each tissue and each subtype. Bar plots (right panel) present the average CPMs of ligand and receptor genes in each tissue and each subtype

In addition to the cartilage, other joint tissues, such as the synovium^25,26^ and subchondral bone,^27^ may also contribute to OA etiology. To map the tissue crosstalk in the OA knee, synovium and subchondral bone samples were collected, and BRB-seq was performed for the same OA patients. After quality control, we obtained transcriptome data from 60 synovium samples (37 patients from C1, 12 patients from C2, and 11 patients from C4) and 65 subchondral bone samples (44 patients from C1, 16 patients from C2 and five patients from C4). To dissect intertissue crosstalk, we analyzed ligand-receptor pairing based on the expression of the complementary ligand and receptor in every other tissue (Supplementary Methods). Thus, nine types of tissue crosstalk including cartilage-cartilage crosstalk (cart-cart), cartilage-synovium crosstalk (cart-syno), cartilage-subchondral bone crosstalk (cart-subc), synovium-cartilage crosstalk (syno-cart), synovium-synovium crosstalk (syno-syno), synovium-subchondral bone crosstalk (syno-subc), subchondral bone-cartilage crosstalk (subc-cart), subchondral bone-synovium crosstalk (subc-syno) and subchondral bone-subchondral bone crosstalk (subc-subc) were analyzed in each OA subtype. The numbers of expressed ligand-receptor pairs for syno-subc (526), subc-syno (474) and subc-subc (441) were largest in C1, and the numbers of expressed ligand-receptor pairs for the other six types of tissue crosstalk were largest in C4 (Fig. 3a–c, left panel). These results showed more tissue crosstalk in C4 than the other subtypes. Then, we calculated the occurrence ratio (OR) for ligand-receptor pairs and identified highly expressed ligand-receptor pairs for each paired tissue (Supplementary Methods). Figure 3a–c summarize the number of crosstalk events with highly expressed ligand-receptor pairs in networks. The numbers of highly expressed ligand-receptor pairs for bidirectional subc-syno (204) and syno-subc (207) were largest in C2 (Fig. 3a–c, right panel).

To characterize the functional features of tissue crosstalk in specific OA subtype, GO enrichment analysis of highly expressed ligand-receptor genes was applied (Supplementary Methods). Some biological processes of tissue crosstalk appeared in all OA subtypes, such as extracellular structure organization, ECM organization and tissue development enriched in subc-cart (Fig. 3d, Supplementary Figs. 3 and 4). Ossification enriched in subc-cart and regulation of osteoblast differentiation enriched in subc-subc were both found in C4 (Fig. 3d) and might contribute to subchondral bone overgrowth, thus leading to the narrowed joint space observed in C4 patients. Furthermore, vasculature development and invasion are critical foundations in osteophyte development and bone remodeling. Considering that vasculature development was enriched in the subc-cart of C2 and the cart-subc of C4 (Supplementary Fig. 4 and Fig. 3d), we speculated that the different directional tissue crosstalk patterns might affect disease progression and clinical symptoms.

Next, we investigated the counts per million (CPMs) of two differentially expressed ligand-receptor pairs. The expression of *ITGA4*, the receptor for the *VCAN* ligand, was downregulated in C1 (Fig. 3e), a condition favoring the ACAN metabolic disorder found in C1. *TNFSF11*, which encodes a member of the tumor necrosis factor (TNF) cytokine family, is a ligand for osteoprotegerin and is a key factor for osteoclast differentiation and activation.^28^ The receptor *TNFSF11A* was highly expressed in the cartilage of C2 and subchondral bone of C4. These results suggested that the imbalanced expression of ligands and receptors between tissues affected tissue crosstalk.

### Clinical features of the OA subtypes

**Fig. 4:**
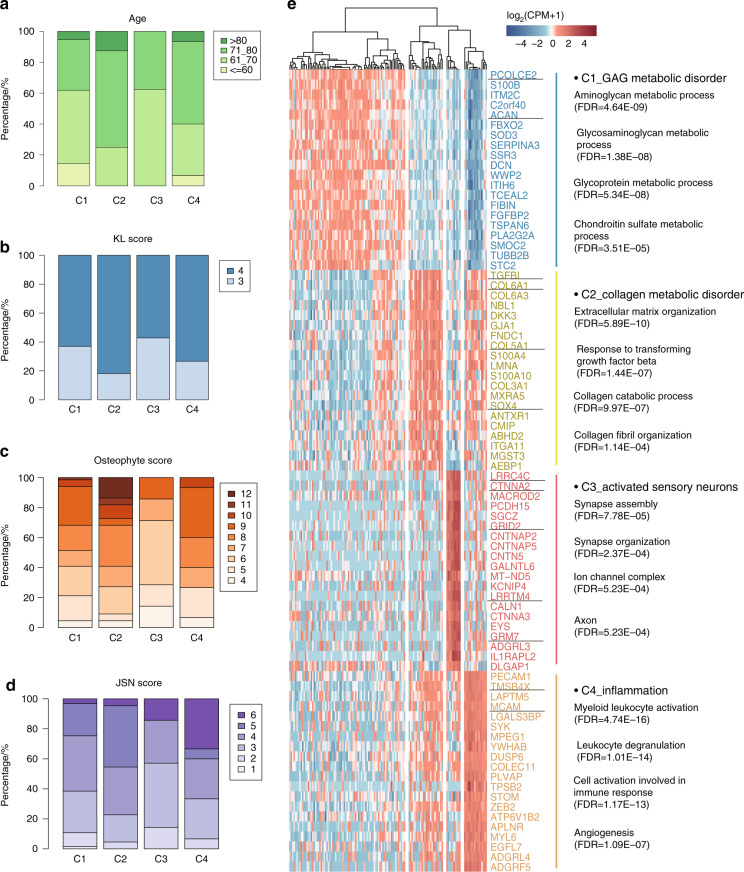
Clinical symptoms and marker genes with specific functions in each OA subtype. **a** Stacked plot of the age percentages in each subtype. **b** Stacked plot of the KL score percentages in each subtype. **c** Stacked plot of the osteophyte score percentages in each subtype. **d** Stacked plot of the joint space narrowing (JSN) score percentages in each subtype. **e** Heatmap showing the expression of the top 20 marker genes ranked by the AUROC in each subtype. The main enriched GO terms of all marker genes in each subtype are listed on the right

Clinical data of the OA patients, including age and Kellgren and Lawrence system (KL), joint space narrowing (JSN) and osteophyte scores, were analyzed for each OA subtype (Supplementary Table 3 and Supplementary Fig. 5). In general, the C3 subtype presented in a greater number of relatively young individuals (Fig. 4a), while 43% of the C3 subtype patients had a KL graded score of 3 (Fig. 4b), suggesting milder symptoms in C3 compared to the other subtypes. In the C2 subtype, 27.5% of the patients had an osteophyte score greater than 10 (Fig. 4c), which was significantly higher than the scores of C1 patients (*P* = 0.035, Cochran-Armitage test, FDR = 0.05). This finding might suggest that osteophytes could be one of the prominent symptoms of C2 OA patients. We also found that 34% of C4 patients had a JSN score of 6 (Fig. 4d), and the JSN score of C4 was significantly higher than that of C1 (*P* = 0.039, Cochran-Armitage test, FDR = 0.12). This suggested that JSN might be a trait in C4 OA patients (detailed distributions are shown in Supplementary Fig. 5, and detailed statistical test results are listed in Supplementary Table 4). The fact that specific OA clinical symptoms could be linked with a corresponding molecular mechanism suggests that our classification method is clinically reasonable. Thus, we next sought to identify potential marker genes for each subtype and validated the subtypes we identified here with independent public datasets.

### Marker genes in the four OA subtypes

Through the SC3^14^ method with an adjusted *P* value less than 0.05, we identified 211 marker genes for C1, 37 marker genes for C2, 86 marker genes for C3, and 230 marker genes for C4. The marker genes for each subtype were ranked by the area under the receiver operating characteristic curve (AUC), and the top 20 genes were visualized in a heatmap (Fig. 4e, Supplementary Methods). From the results, highly expressed genes, including *LRRC4C*, *CTNNA2*, *GRID2*, *LRRTM4*, *GRM7*, and *ADGRL3*, were the top marker genes of C3. The highly expressed gene *ACAN*, which had low expression in C2 and C4, could be a specific marker of C1. The highly expressed genes *COL5A1* and *COL6A1* were identified as marker genes of C2, and *TMSB4X* was identified as a marker gene of C4.

The marker genes of each cluster were enriched in specific GO terms (Fig. 4e). The marker genes of C1 were enriched in the aminoglycan metabolic process, glycosaminoglycan (GAG) metabolic process, chondroitin sulfate metabolic process, and other terms. *PCOLCE2* (also named *PCPE2*), which is expressed in OA cartilage,^29^ was identified as one of the markers of C1. *Pcolce2* expression was found in the interior nonossified regions of cartilaginous structures and excluded from regions of ossification in mice. The distribution of *Pcolce2* expression precisely matches the distribution of the proteoglycans of nonossified cartilage.^30^ The marker genes of C2 were enriched in GO terms such as ECM organization and cell adhesion. Furthermore, two functional terms associated with collagen metabolism, the collagen metabolic process and collagen fibril organization, were also identified in this subtype. TGFβ-induced gene (*TGFBI*) is a marker of C2 that plays a role in cell adhesion and collagen interactions.^31^ The expression of *TGFBI* family members is upregulated in the cartilage and bone of patients with OA, whereas it is downregulated in bone marrow-derived human mesenchymal stem cells (MSCs).^32^ Another marker, *SOX4*, is involved in osteoarthritic cartilage deterioration through the induction of *ADAMTS4* and *ADAMTS5* in the human cell line SW1353.^33^ *PART1* can modulate chondrocyte proliferation, apoptosis, and ECM degradation by sponging *miR-373-3p* and regulating *SOX4* expression in OA.^34^ The marker genes of C4 showed that myeloid leukocyte activation, leukocyte degranulation, and angiogenesis were the most significantly enriched functional terms. Thymosin β4 (*TMSB4X*) is a marker gene of C4 that significantly affects actin polymerization, wound healing, apoptosis, inflammatory responses, and angiogenesis^35^ and activates matrix metalloproteinase (MMP)-2 and MMP9 in the articular cartilage.^36^ Under OA conditions, chondrocytes in the joint cartilage release large amounts of MMPs, which leads to cartilage destruction. The MMPs expressed at increased levels regulate the recruitment and influx of inflammatory cells to the site of inflammation by processing ECM components, growth factors, cytokines, and chemokines.^37^ Consistent with our previous observation, the marker genes of C3 were enriched in synapse assembly. Most of the marker genes of C3 were identified to affect neurogenesis. As an example, *Grm7* knockdown leads to persistent abnormal neuronal development, while *Grm7* overexpression ameliorates the defects in neurogenesis caused by *Grm7* knockdown in mice.^38^ According to the functions of the marker genes of each subtype, these subtypes were named C1-Glycosaminoglycan Metabolic Disorder, C2-Collagen Metabolic Disorder, C3-Activated Sensory Neurons, and C4-Inflammation.

To validate the identified subtypes in our study with an independent dataset, we built a random forest-based classification model using two public OA RNA-seq datasets, E-MTAB-6266^39^ and GSE114007,^17^ as the testing datasets (Supplementary Methods, Supplementary Fig. 6). In the dataset E-MTAB-6266, we identified three subtypes, including 30 C1 samples (69.8%, 30/43), eight C2 samples (18.6%, 8/43) and five C4 samples (11.6%, 5/43). C3 was not identified in the dataset E-MTAB-626, so we analyzed the age distribution of the patients included in the dataset E-MTAB-626. The age distribution showed that the average age of the patients in E-MTAB-626 was 75 years with a standard deviation of 6.2 years (the oldest patient was 85 years old, and the youngest was 63 years old). These results indicated that the patient ages in E-MTAB-626 were older than those in our C3 subtype (average age was 69.2 years with a standard deviation of 5.1 years). This may be the reason why C3 was not identified in the E-MTAB-626 dataset. Another possibility is that the proportion of the C3 subtype is relatively small so C3 might not have been identified given the small sample size of E-MTAB-6266. In the dataset GSE114007, we identified two subtypes including 14 C1 samples (70%, 14/20) and 6 C2 samples (30%, 6/20).

**Fig. 5:**
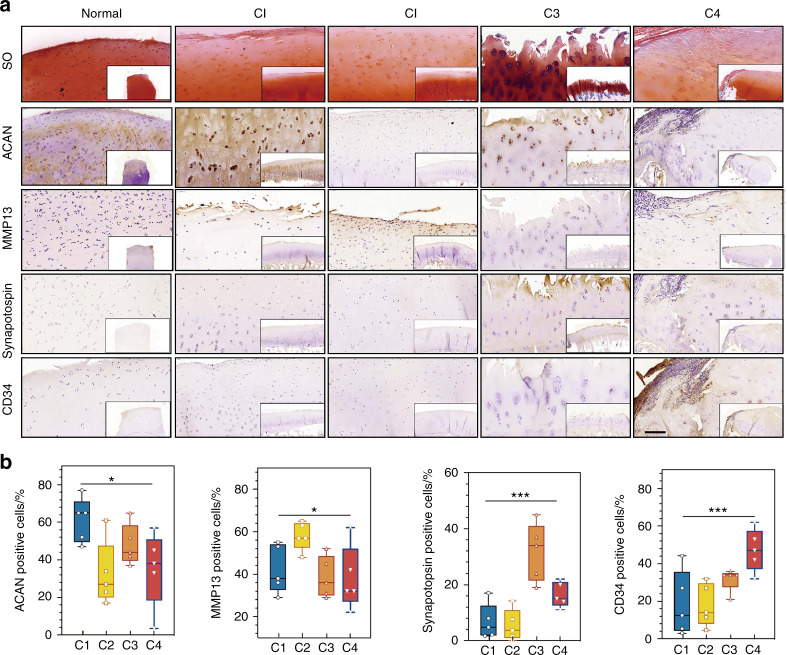
**a** Representative immunohistochemical analysis of the specific pathways in cartilage tissues of each subtype. Scale bar, left, 200 μm; lower right, 400 μm. **b** The percentage of positive cells in each subtype based on the immunohistochemical analysis shown in **a**. *P < 0.05, ***P < 0.001

To validate our OA subtype classification, we performed immunohistochemistry (IHC) to confirm the specific pathways in each subtype (Fig. 5a, b). Compared to normal cartilage samples, all OA samples presented a reduction in the proteoglycan level and a defective cartilage surface indicated by Safranin O staining, confirming their osteoarthritic properties. We further confirmed that the representative genes of specific pathways correlated well with quantitative immunohistochemistry results. Positive staining for ACAN in the articular cartilage was significantly higher in C1 patients, while MMP13-positive cells dominated the collagen metabolic disorder C2 subtype. Synaptophysin (SYP) is related to pathways of the synaptic vesicle cycle,^40^ which was highly detected in patients classified as having the C3 activated sensory neuron subtype. CD34, which is commonly described as a hematopoietic stem cell marker and associated with inflammatory responses, was more enriched in the C4 inflammation subtype.

## Discussion

The current standard bulk transcriptomic workflow utilizes late multiplexing and processes the samples on a one-by-one basis. Learning from single-cell RNA profiling designed for an early multiplexing protocol, BRB-seq provides a great capacity for transforming large sets of tissue samples into a unique sequencing library. Tissue samples are individually labeled, pooled together and then analyzed in bulk to shorten the run time and cost for library preparation and sequencing. Although they have a slightly lower sensitivity than conventional mRNA-seq, 3’ digital gene expression assays are effective for detecting genome-wide gene expression levels with stringent quality control (QC).^41^ The limitation of BRB-seq is the inability to capture low-abundance genes and address RNA splicing and fusion gene research questions. Considering these advantages and disadvantages, BRB-seq is suitable for research with high-abundance genes in large sets of samples, such as organ-specific feature discovery^42^ and disease classification. OA is a heterogeneous disease with distinct pathogenic processes. Many traditional pharmacological therapies, such as acetaminophen and nonsteroidal anti-inflammatory drugs (NSAIDs), may not be effective for treating all OA patients. To date, there is still no approved disease-modifying osteoarthritis drug (DMOAD).^43^ To help address this, in this study, we created a knee joint tissue transcriptome atlas by using BRB-seq in the largest OA cohort studied to date.

In the present study, the classification model was successfully built by transcriptomic analysis and unsupervised clustering of OA cartilage samples, identifying four distinct OA subtypes and their corresponding functional signatures. These OA subtypes showed high correlations with OA symptoms, such as glycosaminoglycan metabolism disorder (typical clinical symptoms), collagen metabolism disorder (osteophytes), activated sensory neurons (perhaps joint pain) and inflammation (a narrowed joint space) (Table 1).

**Table 1:** The features of the four identified OA subtypes

Features	C1: GAG metabolic disorder	C2: Collagen metabolic disorder	C3: Activated sensory neurons	C4: Inflammation
Molecular features	*PCOL CE2/ACAN* ↑ *VCAN* ↓ GAG metabolic ↑	*TFGB/COL6A1* ↑ Collagen catabolic ↑	*GRIK2/GRM7*↑ Synapse assembly ↑	*TMSB4X/CD34* ↑ Immune response ↑
Clinical features	Typical symptom	Severe osteophyte	Younger age of KRS	Apparently narrow joint space
Treatment principles	GAG supplement	Collagen supplement	Analgesia	Anti-inflammation
Drugs	Hyaluronic acid Hyaluronic Glucosamine	Gelatin Hyaluronic collagen Undenatured collagen	NGF-inhibitor (tanezumab) Acetaminophen Opioids	NSAIDs Corticosteroids IL-1 inhibitor (canakinumab) TNF-α inhibitor (adalimumab, infliximab, etanercept)

Briefly, the C1-glycosaminoglycan metabolic disorder OA subtype presented typical clinical symptoms. In this subtype, *ACAN* was highly expressed, and *VCAN* was lowly expressed, suggesting a disordered proteoglycan composition in the joint. The ratio of ACAN to VCAN (*ACAN/VCAN*) is significantly higher in normal cartilage than in OA cartilage.^44^ Unlike the other subtypes, C1 did not present any specific symptoms. Therefore, this subtype may present a relatively earlier stage than the other subtypes. The C2-collagen metabolic disorder OA subtype represented osteophytes in the clinic. Osteophytes may develop from pluripotent chondrocyte differentiation in vivo.^45^ *COL5A1* and *COL6A1*, which have been previously reported to be associated with ossification,^45,46^ were highly expressed in this subtype. Moreover, *COL6A1* and *TGFBI* were identified as two marker genes of C2. According to tissue crosstalk analysis, vasculature development was significantly enriched in the subchondral bone-cartilage crosstalk of C2. These molecular and functional signatures are consistent with the clinical symptoms of osteophytes.

The C3-activated sensory neuron OA subtype, which included the youngest patients, exhibited high expression of neural molecules, such as *LRRC4C*, *CTNNA2*, and *GRID2*, which suggested the pain sensitivity of this subtype. Many genes encoding ion channels, such as *KCNIP4* and *GRM7*, were also highly expressed in this subtype. The increased expression of both neural markers and ion channel remodeling genes may be caused by biomechanical changes in the articular cartilage, promoting pain sensitivity. Notably, the concept of the activated sensory neuron OA group was proposed for the first time in this study. To the best of our knowledge, sensory nerve fibers are observed in contact with a subpopulation of chondrocytes located in the growth cartilage and at the surface of the articular cartilage.^47,48^ Calcitonin gene-related peptide (CGRP)-positive fibers, which originate from the periosteum and near insertion regions of the muscles and tendons, innervate up to 25 μm into the articular and meniscal cartilage tissues in rat knee joints.^47^ Furthermore, Suri et al. localized both sensory (SP- and CGRP-positive) and sympathetic [neuropeptide Y (NPY)-positive] nerve fibers in the articular cartilage in human tibiofemoral OA, and both of these nerve fiber types were present within vascular channels in both mild and severe OA stages.^49^ According to these findings, the nerves are localized in the perivasculature in the surface layer of the cartilage. Therefore, vascularization leads to innervation. In addition, perivascular nerve growth may contribute to pain in OA because nerve growth is associated with peripheral sensitization and nerves in structures that are generally not innervated, such as cartilage, could be exposed to chemical stimulation and mechanical stress. Thus, neovascularization may contribute to pain in patients with OA because of the accompanying sensory innervation.^50^ We proposed that the abundance of sensory nerves is the driving force of OA pain. The pathogenesis of C3 gives rise to patients choosing knee replacement surgery (KRS) at a relatively young age.

The C4-inflammation OA subtype presented high inflammation and a narrow joint space in the clinic. The marker genes *TMSB4X* and *CD34* were highly expressed in the cartilage, and the protein SDF1α, which actives T lymphocytes and monocytes but not neutrophils,^51,52^ was highly expressed, whereas IL10 (an inhibitory cytokine)^53^ was expressed at low levels in the synovial fluid. These features promoted the high inflammation observed in this subtype. According to the results of joint tissue crosstalk analysis, the function of ossification was enriched in subchondral bone-cartilage crosstalk, the function of osteoblast differentiation regulation was enriched in subchondral bone-subchondral bone crosstalk, and the function of vasculature development was enriched in cartilage-subchondral bone crosstalk. These results may be the cause of the narrow joint space characteristic of C4.

Here, we listed some potential treatments for the four OA subgroups. Injection of members of the glycosaminoglycan family, including hyaluronic acid, chondroitin, and glucosamine, provides therapeutic benefits for OA due to chondroprotective effects.^54^ These “chondroprotectants” should be the first option for people in the C1 subgroup. The treatments for patients in the C2 subgroup should include collagen supplements, such as gelatin, hydrolyzed collagen, and undenatured collagen. In particular, pharmaceutical-grade collagen hydrolysate (PCH) and undenatured type II collagen (UC-II) have been shown to improve the symptoms of OA by stimulating joint collagen.^55,56^ On the other hand, nerve growth factor (NGF) (a neurotrophin) is necessary for the normal development of the sympathetic nervous system and sensory neurons. For individuals with musculoskeletal pain, treatment with NGF inhibitors such as tanezumab, fulranumab, and fasinumab can produce significant improvements in joint pain and physical function, which may be a potent analgesic for patients with C3-subtype OA.^57^ For patients in the C4 subgroup, the primary approach is the application of anti-inflammatory agents. Hence, NSAIDs, corticosteroids, and TNF and IL-1 inhibitors are highly recommended for C4-subgroup patients.^58–60^ Monotherapies targeting one or a few pathogenic mechanisms have not been effective in treating all OA patients. In the future, the development of effective DMOADs will be feasible and practicable if specific OA subgroups are precisely targeted through the combination of clinical data and molecular features.

## Materials and methods

For full methods, see the Supplementary Methods.

### Participants, samples and transcriptome library preparation

The selected patients fulfilled the American College of Rheumatology classification criteria for OA and had no history of knee injury, surgery, rheumatoid arthritis or pseudogout. OA was confirmed in patient knee joint X-ray images. Samples of OA cartilage, subchondral bone, synovium, and synovial fluid were obtained from patients undergoing knee replacement surgery at four clinical research centers in China. Cartilage tissues obtained from individuals undergoing amputation (without OA) were used as control samples. A total of 232 cartilage samples were collected (227 OA samples and five amputation samples without OA). The whole layer of the cartilage around the full-thickness cartilage defect was selected to create samples from the central area of the medial femoral condyle (Supplementary Fig. 7). For this study, subchondral trabecular bone cores (0.5 cm in diameter and 1 cm in depth) were obtained from areas immediately underlying the previously examined eroded cartilage using a surgical trephine. In total, we obtained 227 OA cartilage samples, five control cartilage samples, 60/227 OA synovium samples and 65/227 OA subchondral bone samples. Among the 227 collected knee samples, 54 sets of the cartilage, synovium and subchondral bone samples were from the same individuals. Informed consent was obtained from all participants. This study was approved by the local clinical ethics committees of Zhejiang University School of Medicine and the affiliated hospital of Wenzhou Medical University (2017KYLL11). RNA extraction and RNA-seq library preparation of the cartilage, synovium and subchondral bone samples were performed using the BRB-seq^13,42^ protocol (details in the Supplementary Methods).

### Clinical scores

The KL is a commonly used five-grade measurement to grade knee OA severity.^61^ KL graded scores were either 3 or 4 in all the study cohorts. For osteophyte symptoms, we used a baseline posterior-anterior (PA) radiograph and scored each marginal osteophyte on a scale of 0–3 according to the Osteoarthritis Research Society International (OARSI) atlas.^62^ For each knee, we scored four sites: The medial tibia, medial femur, lateral tibia, and lateral femur. The osteophyte score was the sum of the scores of the four sites. According to the OARSI atlas, JSN was evaluated in the lateral part and medial part of the femoral tibia. Each part was divided into 0–3 grades.^62^ The total knee rating corresponded to the sum of the rating scores of both parts. Detailed deidentified patient information is listed in Supplementary Table 3.

### RNA-seq data analysis and unsupervised clustering

FASTX-Toolkit version 0.0.13 (http://hannonlab.cshl.edu/fastx_toolkit/commandline.html; FASTQ Quality Filter function) was used for quality control of raw RNA-seq reads. Reads would be retained if greater than 80% (parameter *p* = 80) of bases within the reads has a minimum quality score of 19 (parameter –*q* = 19). The reads were then aligned to the GENECODE hg38.p5 human reference genome using STAR with the default setting.^63^ Counts for all genes were calculated using featureCounts v1.6.0^64^ with default settings. To avoid bias introduced by sequencing depth variation, samples with total counts lower than 500 000 or total counts higher than 2 000 000 were excluded. After this step, 131 OA samples and four control samples were kept for the following analysis. The expression level of each gene was then normalized using the CPM. To minimize noise effects, we discarded genes with an expression variance higher than three based on control samples without OA. We also kept only genes with a CPM larger than five in at least 15 OA samples. This normalized count matrix was then utilized to identify OA clusters with the unsupervised clustering method single-cell consensus clustering (sc3)^14^ by combining multiple clustering solutions in the R/Bioconductor package “SC3”, which was used to process barcoded single-cell RNA-seq datasets with similar dimensions. Genes with the top n highest standard deviations were considered highly variable genes (we tried *n* equal to 2 500, 3 000, 3 500, 4 000, 4 500, and 5 000). To select an appropriate parameter *k* (the SC3 parameter *k*-means, used in the *k*-means and hierarchical clustering), we tested *k* from 2 to 8 iteratively. For each SC3 run with different *k* values, the silhouette score was calculated, the consensus matrix was plotted, and cluster-specific genes were identified. In combination, all three aspects helped us determine the optimal *k* and *n*. Ultimately, we found that the average silhouette score was largest when variable *n* = 4 000 and *k*-means = 3 (Supplementary Fig. 8a). Additionally, we found one subcluster separated into two clusters when *k*-means = 4 (Supplementary Fig. 8b). We discussed all the clustering results with clinical doctors. The clinical doctors supported the clustering of four OA subtypes that were perfectly relevant to clinical symptoms. Thus, we used *n* = 4 000 and *k*-means = 4, which was the best parameter. The detailed clustering results are listed in Supplementary Table 3.

### Statistical analysis

To assess discrepancies in clinical scores, the Cochran–Armitage test was used to evaluate the pairwise significance of clinical symptoms between every two subtypes. Fisher’s exact test was used to compare the significance of sex among subtypes. The Wilcoxon rank-sum test was used to calculate the significance of secreted proteins in the synovial fluid across the subtypes and to measure the significance of the fractions of CD8^+^ T cells and neutrophils across three subtypes. The FDR was used for multiple-inspection corrections in all multiple tests. All statistics are shown in Supplementary Table 4.

## Supplementary information

Supplementary Method

Supplementary Figure 1

Supplementary Table 1

Supplementary Figure 2

Supplementary Table 2

Supplementary Figure 3

Supplementary Figure 4

Supplementary Table 3

Supplementary Figure 5

Supplementary Table 4

Supplementary Figure 6

Supplementary Figure 7

Supplementary Figure 8

## Data Availability

All data from the study are available in online supplementary files. All RNA-seq and metadata are available at SRA (PRJNA505578) or GitHub: https://github.com/Ellen1101/OA-subtype.

## References

[1] Lawrence RC (2008). Estimates of the prevalence of arthritis and other rheumatic conditions in the United States. Part II. Arthritis. Rheum..

[2] Goldring MB, Goldring SR (2010). Articular cartilage and subchondral bone in the pathogenesis of osteoarthritis. Ann. NY Acad. Sci..

[3] Loeser RF, Collins JA, Diekman BO (2016). Ageing and the pathogenesis of osteoarthritis. Nat. Rev. Rheumatol..

[4] Beyer C (2015). Signature of circulating microRNAs in osteoarthritis. Ann. Rheum. Dis..

[5] Glyn-Jones S (2015). Osteoarthritis. Lancet.

[6] Bennell KL, Hall M, Hinman RS (2016). Osteoarthritis year in review 2015: rehabilitation and outcomes. Osteoarthr. Cartil..

[7] Altman R (1986). Development of criteria for the classification and reporting of osteoarthritis. Classification of osteoarthritis of the knee. Diagnostic and Therapeutic Criteria Committee of the American Rheumatism Association. Arthritis. Rheum..

[8] Kraus VB (2017). Predictive validity of biochemical biomarkers in knee osteoarthritis: data from the FNIH OA Biomarkers Consortium. Ann. Rheum. Dis..

[9] Junker S (2016). Differentiation of osteophyte types in osteoarthritis - proposal of a histological classification. Joint Bone Spine.

[10] Farmer H (2005). Targeting the DNA repair defect in BRCA mutant cells as a therapeutic strategy. Nature.

[11] Perou CM (2000). Molecular portraits of human breast tumours. Nature.

[12] Martin JA, Buckwalter JA (2002). Aging, articular cartilage chondrocyte senescence and osteoarthritis. Biogerontology.

[13] Alpern D (2019). BRB-seq: ultra-affordable high-throughput transcriptomics enabled by bulk RNA barcoding and sequencing. Genome Biol..

[14] Kiselev VY (2017). SC3: consensus clustering of single-cell RNA-seq data. Nat. Methods.

[15] Luo W, Friedman MS, Shedden K, Hankenson KD, Woolf PJ (2009). GAGE: generally applicable gene set enrichment for pathway analysis. BMC Bioinform..

[16] Appleton CT, Pitelka V, Henry J, Beier F (2007). Global analyses of gene expression in early experimental osteoarthritis. Arthritis. Rheum..

[17] Fisch KM (2018). Identification of transcription factors responsible for dysregulated networks in human osteoarthritis cartilage by global gene expression analysis. Osteoarthr. Cartil..

[18] Love MI, Huber W, Anders S (2014). Moderated estimation of fold change and dispersion for RNA-seq data with DESeq2. Genome Biol..

[19] Wu G, Haw R (2017). Functional interaction network construction and analysis for disease discovery. Methods Mol. Biol..

[20] Qin Y, Zhang C (2017). The regulatory role of IFN-gamma on the proliferation and differentiation of hematopoietic stem and progenitor cells. Stem Cell Rev..

[21] Kapoor M, Martel-Pelletier J, Lajeunesse D, Pelletier JP, Fahmi H (2011). Role of proinflammatory cytokines in the pathophysiology of osteoarthritis. Nat. Rev. Rheumatol..

[22] El Kasmi KC (2007). Cutting edge: a transcriptional repressor and corepressor induced by the STAT3-regulated anti-inflammatory signaling pathway. J Immunol.

[23] Ally A (2017). Comprehensive and integrative genomic characterization of hepatocellular carcinoma. Cell.

[24] Newman AM (2015). Robust enumeration of cell subsets from tissue expression profiles. Nat. Methods.

[25] Siebuhr AS (2016). Inflammation (or synovitis)-driven osteoarthritis: an opportunity for personalizing prognosis and treatment?. Scand. J. Rheumatol..

[26] Rahmati M, Mobasheri A, Mozafari M (2016). Inflammatory mediators in osteoarthritis: a critical review of the state-of-the-art, current prospects, and future challenges. Bone.

[27] Mahjoub M, Berenbaum F, Houard X (2012). Why subchondral bone in osteoarthritis? The importance of the cartilage bone interface in osteoarthritis. Osteoporos Int..

[28] Teitelbaum SL, Ross FP (2003). Genetic regulation of osteoclast development and function. Nat. Rev. Genet..

[29] Kumar S (2001). Identification and initial characterization of 5000 expressed sequenced tags (ESTs) each from adult human normal and osteoarthritic cartilage cDNA libraries. Osteoarthritis Cartilage.

[30] Steiglitz BM, Keene DR, Greenspan DS (2002). PCOLCE2 encodes a functional procollagen C-proteinase enhancer (PCPE2) that is a collagen-binding protein differing in distribution of expression and post-translational modification from the previously described PCPE1. J. Biol. Chem..

[31] Skonier J (1994). beta ig-h3: a transforming growth factor-beta-responsive gene encoding a secreted protein that inhibits cell attachment in vitro and suppresses the growth of CHO cells in nude mice. DNA Cell Biol..

[32] Ruiz M (2019). TGFbetai is involved in the chondrogenic differentiation of mesenchymal stem cells and is dysregulated in osteoarthritis. Osteoarthr. Cartil..

[33] Takahata Y (2019). Sox4 is involved in osteoarthritic cartilage deterioration through induction of ADAMTS4 and ADAMTS5. FASEB J.

[34] Zhu YJ, Jiang DM (2019). LncRNA PART1 modulates chondrocyte proliferation, apoptosis, and extracellular matrix degradation in osteoarthritis via regulating miR-373-3p/SOX4 axis. Eur. Rev. Med. Pharmacol. Sci..

[35] Goldstein AL, Hannappel E, Sosne G, Kleinman HK (2012). Thymosin beta4: a multi-functional regenerative peptide. Basic properties and clinical applications. Expert Opin Biol Ther.

[36] Blain EJ, Mason DJ, Duance VC (2002). The effect of thymosin beta4 on articular cartilage chondrocyte matrix metalloproteinase expression. Biochemical. Soc. Trans..

[37] Nissinen L, Kahari VM (2014). Matrix metalloproteinases in inflammation. Biochim. Biophys. Acta.

[38] Xia W, Liu Y, Jiao J (2015). GRM7 regulates embryonic neurogenesis via CREB and YAP. Stem Cell Rep..

[39] Soul, J. et al. Stratification of knee osteoarthritis: two major patient subgroups identified by genome-wide expression analysis of articular cartilage. *Ann. Rheum. Dis.* **77**, 423 (2018). 10.1136/annrheumdis-2017-212603 PMC5867416 29273645

[40] Sudhof TC (2004). The synaptic vesicle cycle. Annu. Rev. Neurosci..

[41] Xiong Y (2017). A comparison of mRNA sequencing with random primed and 3’-directed libraries. Sci. Rep..

[42] Wu B (2019). Nano genome altas (NGA) of body wide organ responses. Biomaterials.

[43] Van Spil WE, Kubassova O, Boesen M, Bay-Jensen AC, Mobasheri A (2019). Osteoarthritis phenotypes and novel therapeutic targets. Biochem. Pharmacol..

[44] Martin I (2001). Quantitative analysis of gene expression in human articular cartilage from normal and osteoarthritic joints. Osteoarthr. Cartil..

[45] Gelse K, Soder S, Eger W, Diemtar T, Aigner T (2003). Osteophyte development—molecular characterization of differentiation stages. Osteoarthr. Cartil..

[46] Kahai S, Vary CP, Gao Y, Seth A (2004). Collagen, type V, alpha1 (COL5A1) is regulated by TGF-beta in osteoblasts. Matrix Biol..

[47] Schwab W, Funk RH (1998). Innervation pattern of different cartilaginous tissues in the rat. Acta Anat..

[48] Grässel, S. & Muschter, D. Peripheral nerve fibers and their neurotransmitters in osteoarthritis pathology. *Int. J. Mol. Sci.* **18**, 931 (2017). 10.3390/ijms18050931 PMC5454844 28452955

[49] Suri S (2007). Neurovascular invasion at the osteochondral junction and in osteophytes in osteoarthritis. Ann. Rheum. Dis..

[50] Ashraf S (2011). Increased vascular penetration and nerve growth in the meniscus: a potential source of pain in osteoarthritis. Ann. Rheum. Dis..

[51] Malik M (2008). Monocyte migration and LFA-1-mediated attachment to brain microvascular endothelia is regulated by SDF-1 alpha through Lyn kinase. J. Immunol..

[52] Oberlin E (1996). The CXC chemokine SDF-1 is the ligand for LESTR/fusin and prevents infection by T-cell-line-adapted HIV-1. Nature.

[53] Ip WKE, Hoshi N, Shouval DS, Snapper S, Medzhitov R (2017). Anti-inflammatory effect of IL-10 mediated by metabolic reprogramming of macrophages. Science.

[54] Zhang, Y., Chen, X., Tong, Y., Luo, J. & Bi, Q. Development and Prospect of Intra-Articular Injection in the Treatment of Osteoarthritis: A Review. *J. Pain. Res*. **13**, 1941–1955 (2020). 10.2147/JPR.S260878 PMC7414982 32801850

[55] Moskowitz RW (2000). Role of collagen hydrolysate in bone and joint disease. Semin. Arthritis. Rheum..

[56] Lugo JP, Saiyed ZM, Lane NE (2016). Efficacy and tolerability of an undenatured type II collagen supplement in modulating knee osteoarthritis symptoms: a multicenter randomized, double-blind, placebo-controlled study. Nutr. J..

[57] Lane NE, Corr M (2017). Osteoarthritis in 2016: Anti-NGF treatments for pain - two steps forward, one step back?. Nat. Rev. Rheumatol..

[58] Conaghan PG, Cook AD, Hamilton JA, Tak PP (2019). Therapeutic options for targeting inflammatory osteoarthritis pain. Nat. Rev. Rheumatol..

[59] da Costa BR (2017). Effectiveness of non-steroidal anti-inflammatory drugs for the treatment of pain in knee and hip osteoarthritis: a network meta-analysis. Lancet.

[60] da Costa BR, Hari R, Jüni P (2016). Intra-articular corticosteroids for osteoarthritis of the knee. JAMA.

[61] Kellgren JH, Lawrence JS (1957). Radiological assessment of osteo-arthrosis. Ann. Rheum. Dis..

[62] Altman RD, Gold GE (2007). Atlas of individual radiographic features in osteoarthritis, revised. Osteoarthr. Cartil..

[63] Dobin A (2013). STAR: ultrafast universal RNA-seq aligner. Bioinformatics.

[64] Liao Y, Smyth GK, Shi W (2014). featureCounts: an efficient general purpose program for assigning sequence reads to genomic features. Bioinformatics.

