# Supplementary material for: Classification of four distinct osteoarthritis subtypes with a knee joint tissue transcriptome atlas
Source: Bone Res. 2020 Nov 12;8:38. doi: 10.1038/s41413-020-00109-x (PMC7658991; doi:10.1038/s41413-020-00109-x)
Supplement: Supplementary file 1 — Supplementary Method [file 41413_2020_109_MOESM1_ESM.docx]

**Supplementary Method**

**RNA extraction and reverse transcription**

Total RNA was isolated from homogenized cartilage, synovia and subchondral bone samples using the RNeasy Fibrous Tissue Mini Kit (Qiagen, Duesseldorf, Germany) following the manufacturer’s instructions. The RNA concentration and purity were determined using Nano-Drop (NanoDrop Technologies, DE, USA). The integrity of the RNA was assessed in a Bioanalyzer 2100 (Agilent Technologies, CA, USA) so that only those samples with a RIN (RNA integrity number) value equal or greater than 7 were used.

Total RNA was mixed with 1μl of anchored oligo-dT primer and 1 μl of dNTP mix (10 mM, Fermentas), denatured at 72 °C for 5 min. Seven microliters of the first-strand reaction mix (containing 0.50 μl SuperScript II reverse transcriptase (Invitrogen), 0.25 μl RNAse inhibitor (Clontech), 2 μl Superscript II First-Strand Buffer (Invitrogen), 0.25 μl DTT (Invitrogen), 1 μl betaine (Sigma), 0.9 μl MgCl_2_ (Sigma), and 0.1 μl nuclease-free water (Gibco)) were added to each sample. Reverse transcription reactions were carried out at 42 °C for 90 min, followed by 10 cycles of (50 °C for 2 min, 42 °C for 2 min). The reverse transcriptase was inactivated at 70 °C for 15 min.

The second strand synthesis was carried out after adding 2 μl 10X second strand synthesis buffer, 1 μl Enzyme mix, 100 ng cDNA and water to a final reaction volume of 20 μl. The reaction was incubated at 16 °C for 150 min and then purified with AMPure XP beads.

**Transcriptome libraries preparation and sequencing**

Five nanograms of cDNA were used for the tagmentation reaction using the Nextera DNA Sample Preparation kit (Illumina), with the addition of 2.5 μl 2× Tagment DNA Buffer and 1.25 μl Tagment DNA Enzyme, in a final volume of 5 μl. The tagmentation reaction was incubated at 55 °C for 10 min. The whole volume was then used for limited-cycle enrichment PCR, with 3.75 μl Nextera PCR Primer Mix (NPM), 1.25 μl Index1 primers (N7), and 1.25 μl PCR Primer Cocktail (PPC). A second amplification round was performed as follows: 72 °C 3 min, 98 °C 30 s, then 5 cycles of (98 °C 1 0s, 63 °C 30 s, 72 °C 3 min). Purification was carried out with a 1:1 ratio of AMPure XP beads and samples were loaded on a High-Sensitivity DNA chip to check the quality of the library, while quantification was assayed using Qubit High-Sensitivity DNA kits (Invitrogen). Libraries were diluted to a final concentration of 2 nM and pooled, and 10 pmol were sequenced on Illumina HiSeq 2500. Cartilage samples were sequenced in multiplex, with approximately three lanes. The average sequencing depth was 2.32 million reads per sample (range 0.21 to 17.16 million).

The same processing methods were applied in subchondral bone and synovium. The average sequencing depth of subchondral bone was 4.17 million reads per sample (range 0.21 to 31.04 million). The average sequencing depth of synovium was 1.88 million reads per sample (range 0.13 to 9.79 million).

**Generally Applicable Gene-set Enrichment analysis**

Generally Applicable Gene-set Enrichment (GAGE) ^1^ is applicable independently of RNA-Seq data attributes including sample sizes, experimental designs, assay platforms, and other types of heterogeneity, and consistently achieves superior performance over other frequently used methods. Gene set enrichment was calculated using the R/Bioconductor package “gage”^1^ with functional terms from human go ontology as gene sets in each subgroup. Enriched GO terms were determined by comparing each subgroup against the others with a significance cutoff of FDR < 0.05. Gage uses stat.mean which is the average fold change of per gene set to indicates the direction of the changes. The results of gage were plotted by ggplot2.

**Differentially expressed gene**

To identify differentially expressed genes (DEGs) among the metabolic subgroups, we used R/Bioconductor package “DEseq2”^2^ on gene-level fragment counts. For DE analysis, gene expression was compared between each paired subgroup. We calculated the numbers of DEGs with a cut-off FDR<=0.05 and log2(Fold Chang(FC)) >=2, and the numbers were illustrated by R package “VennDiagram”^3^. We used the Reactome pathway database to construct functional interaction (FI) networks to evaluate the functions of DEGs in each cluster.

**Ligand-receptor pair connectivity mapping**

The list of ligand-receptor pairs from a recent study^4^ (containing 2,557 ligand-receptor pairs) was applied. In each cluster, we identified ligand-receptor pairs expressed in tissue X-tissue Y communication by two steps: (1) Both of the CPM of ligand and receptor were higher than 30 in tissue X and tissue Y. (2) The ligand-receptor pairing in step1 was expressed in more than 25% samples. At last, we count the number of expressed ligand-receptor pairs of tissues crosstalk in each cluster.

To characterized the potential communication of clusters, we compared different ligand-receptor pairs and their functions between every two clusters. Base on the number of expressed ligand-receptor pairs, we calculated the occurrence ratio (OR) for ligand-receptor pairs of tissue-crosstalk in each cluster using the following algorithm. For each ligand-receptor pairs L of tissue X-tissue Y crosstalk in cluster A: OR= (the number of L of tissue X-tissue Y in cluster A / the number of samples containing tissue X-tissue Y in cluster A) / (the number of L of tissue X-tissue Y in all clusters / the number of samples containing tissue X-tissue Y in all clusters). Ligand-receptor pair with log2(OR)>1 was considered as a high expressed ligand-receptor pair of tissue X-tissue Y in cluster A. So that, we had identified all highly expressed ligand-receptor pair of all tissue-crosstalks in all clusters and characterized them functions by R package “clusterprofile”^5^ with p-value<=0.01 and q-value<=0.05. BgRatio is M/N where M is the size of the gene-set annotated to the GO node and N is the total number of genes in the background. GeneRatio is k/n where k is the number of genes within that list n, which are annotated to the GO node, n is the size of the list of genes. Enrichment score measured the degree of enrichment in the GO node which was calculated by GeneRatio/BgRatio.

**Marker genes in each subgroup**

Marker genes in each subgroup were identified by SC3^6^ with the adjusted p-value<0.05. The functional enrichment analysis of marker genes in each subgroup was performed using enrichGO in R package “clusterprofile” with default parameters.

**Classification with random forest model**

Random forest machine learning algorithm is called an ensemble method that combines the predictions of several classifiers to achieve improved prediction accuracy. Here we used the random forest method to build a classification model. Firstly, the marker genes in the specific functional GO term of each subgroup were selected as features to training the classification model. Then, the model was trained with the 2000 trees and a number of predictors/3 as the maximum tree depth. We used three-fold cross-validation to make sure the model is stable. Models were built using the R package randomForest^7^ and ROC curves (supplementary figure 7) were plotted by ROCR^8^. At last, two public datasets, E-MTAB-6266^9^ and GSE114007^10^, were identified to applying the model.

**Immunohistochemistry assay**

Cartilage paraffin sections (6 mm) were incubated with 0.5% (w/v) pepsin (Sangon Biotech, Shanghai, China) in 5 mM HCl at 37 oC for 30 min for antigen retrieval. Endogenous peroxidase was blocked by incubation with 3% (v/v) hydrogen peroxide in methanol for 10min. Nonspecific protein binding was blocked by incubation with 1% (w/v) BSA. Sections were incubated over-night at 4 oC with primary antibodies: rabbit anti-ACAN (1:100; Abcam, ab186414), rabbit anti-MMP13 (1:200; Abcam, ab39012), rabbit anti-Synaptophysin (1:100; Abcam, ab32127), rabbit anti-CD34 (1:100; Abcam, ab81289). After washing off the excess primary antibodies, these samples were incubated with secondary antibodies and incubated the section for 1 h at room temperature (RT). DAB detection system (Solarbio, Cat. #DA1010) were used to visualized the sections. For quantitative analysis, 3 sections from different samples were selected for each group, and the positive/total cell ratio were calculated for each section.

**Reference**

1. Luo W, Friedman MS, Shedden K, Hankenson KD, Woolf PJ. GAGE: generally applicable gene set enrichment for pathway analysis. BMC bioinformatics 2009; 10: 161.

2. Love MI, Huber W, Anders S. Moderated estimation of fold change and dispersion for RNA-seq data with DESeq2. Genome biology 2014; 15(12): 550.

3. Chen H, Boutros PC. VennDiagram: a package for the generation of highly-customizable Venn and Euler diagrams in R. BMC bioinformatics 2011; 12: 35.

4. Ramilowski JA, Goldberg T, Harshbarger J, et al. A draft network of ligand-receptor-mediated multicellular signalling in human. Nat Commun 2015; 6: 7866.

5. Yu G, Wang LG, Han Y, He QY. clusterProfiler: an R package for comparing biological themes among gene clusters. OMICS 2012; 16(5): 284-7.

6. Kiselev VY, Kirschner K, Schaub MT, et al. SC3: consensus clustering of single-cell RNA-seq data. Nature methods 2017; 14(5): 483-6.

7. Svetnik V, Liaw A, Tong C, Culberson JC, Sheridan RP, Feuston BP. Random forest: a classification and regression tool for compound classification and QSAR modeling. J Chem Inf Comput Sci 2003; 43(6): 1947-58.

8. Sing T, Sander O, Beerenwinkel N, Lengauer T. ROCR: visualizing classifier performance in R. Bioinformatics 2005; 21(20): 3940-1.

9. Soul J, Dunn SL, Anand S, et al. Stratification of knee osteoarthritis: two major patient subgroups identified by genome-wide expression analysis of articular cartilage. Ann Rheum Dis 2017.

10. Fisch KM, Gamini R, Alvarez-Garcia O, et al. Identification of transcription factors responsible for dysregulated networks in human osteoarthritis cartilage by global gene expression analysis. Osteoarthritis Cartilage 2018; 26(11): 1531-8.
