# Supplementary figures and images for: Classification of four distinct osteoarthritis subtypes with a knee joint tissue transcriptome atlas
Source: Bone Res. 2020 Nov 12;8:38. doi: 10.1038/s41413-020-00109-x (PMC7658991; doi:10.1038/s41413-020-00109-x)

## genes in GO synapse term

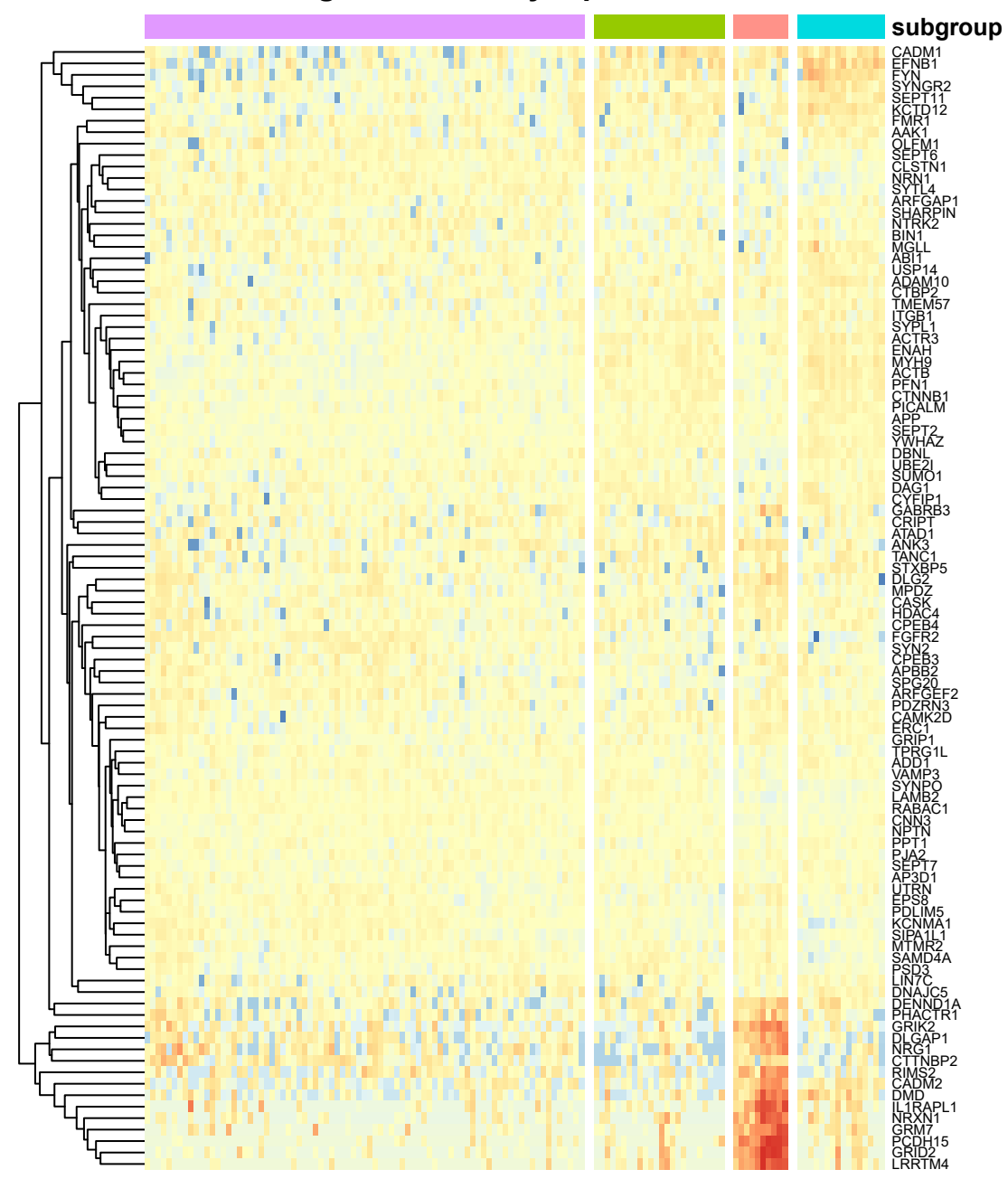

**genes in GO angiogenesis term**

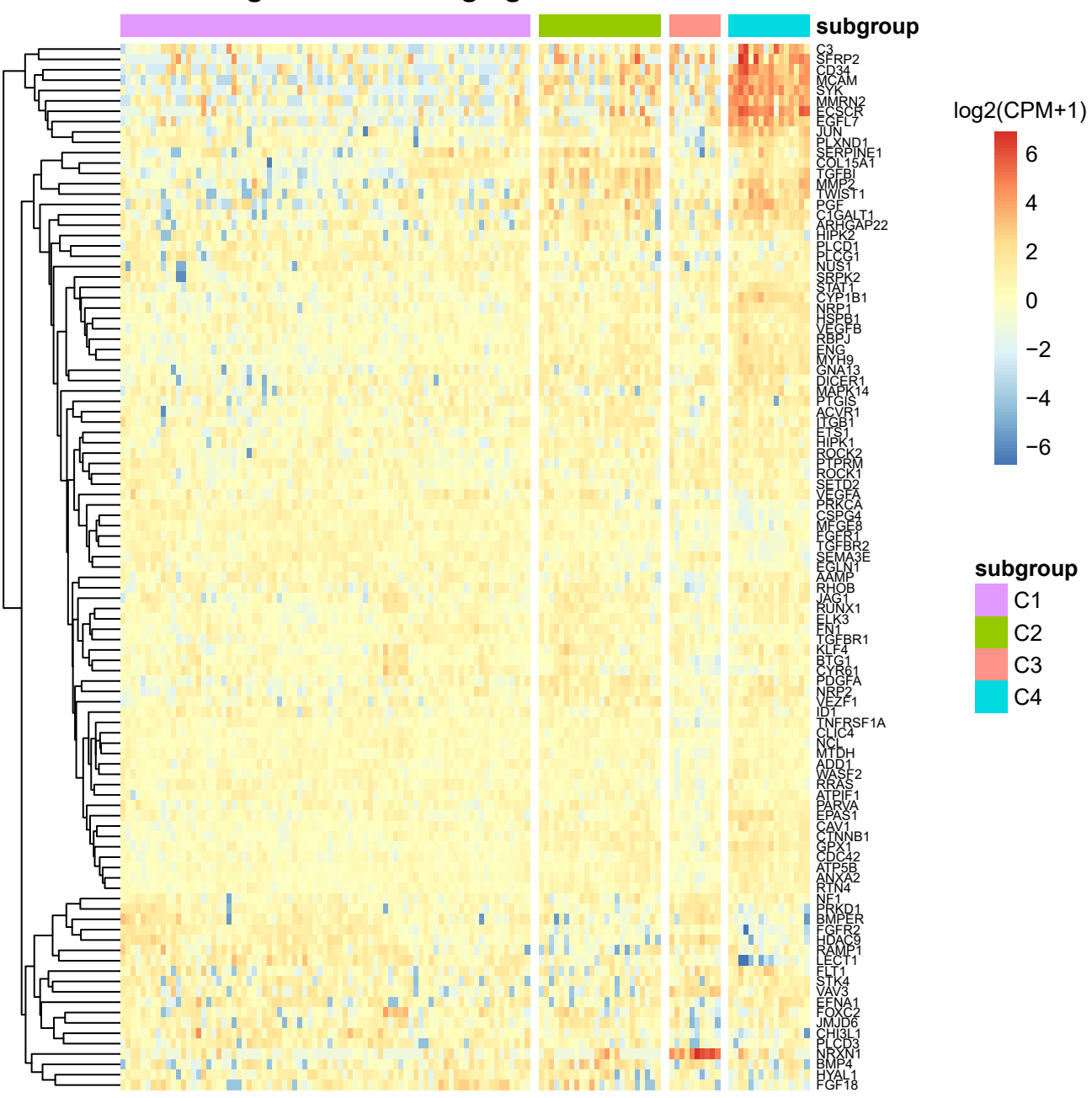

Supplement: Supplementary file 2 — Supplementary Figure 1 [file 41413_2020_109_MOESM2_ESM.pdf]

# tissues corsstalk in C1

GO Description

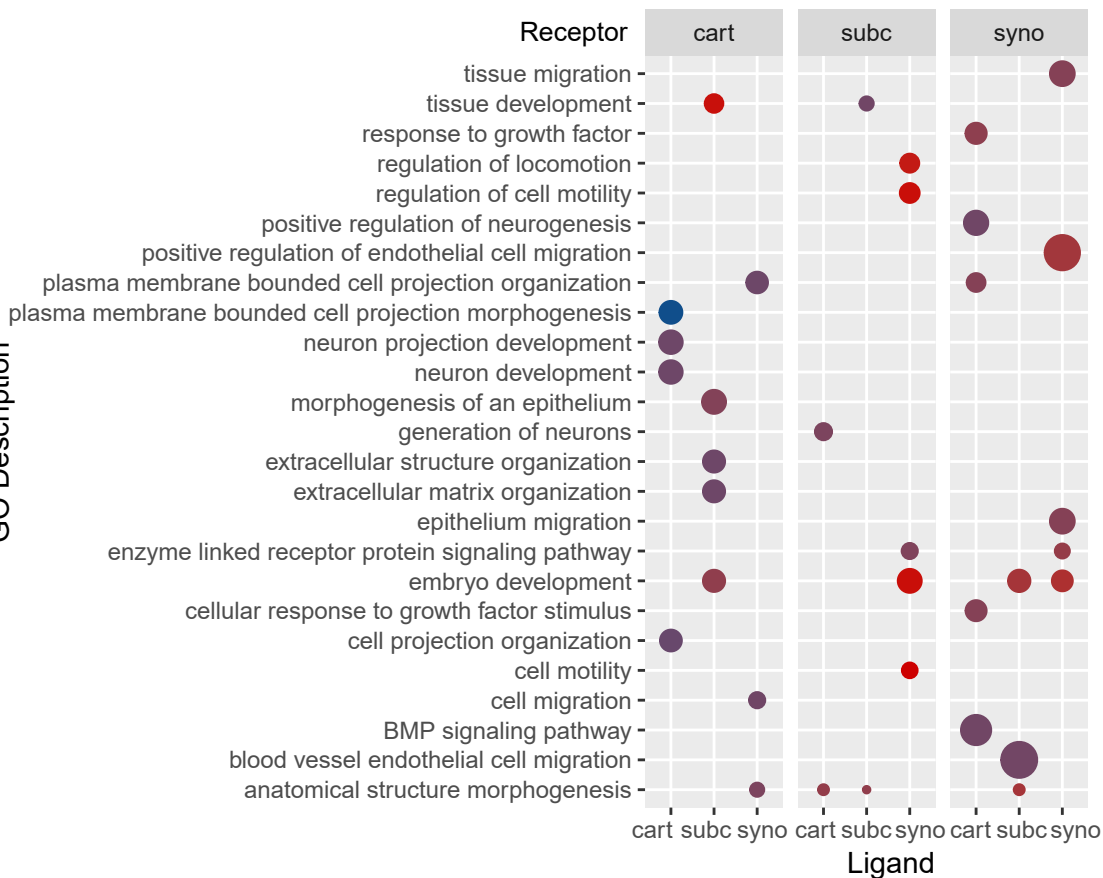

Supplement: Supplementary file 6 — Supplementary Figure 3 [file 41413_2020_109_MOESM6_ESM.pdf]

**ROC curve**

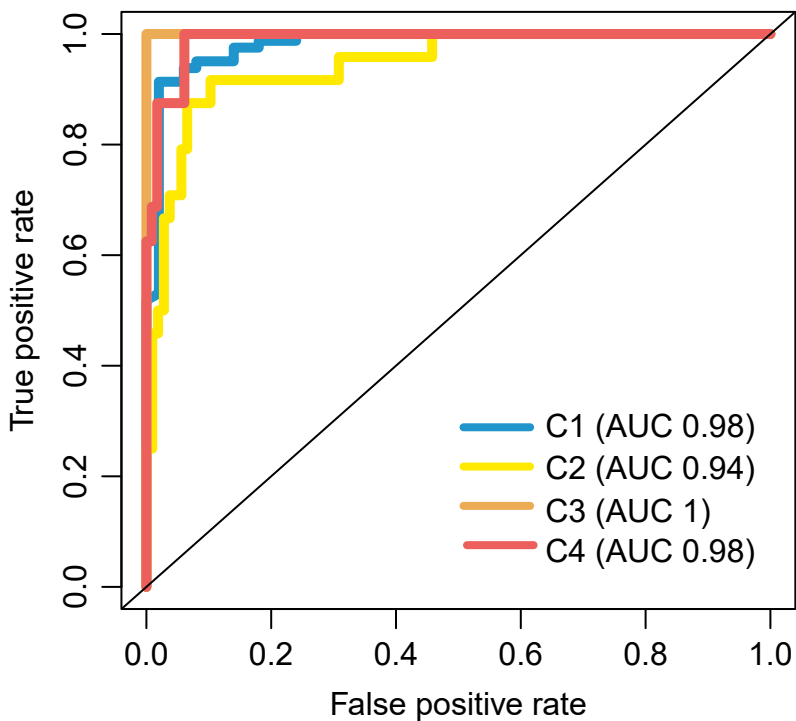

Supplement: Supplementary file 11 — Supplementary Figure 6 [file 41413_2020_109_MOESM11_ESM.pdf]

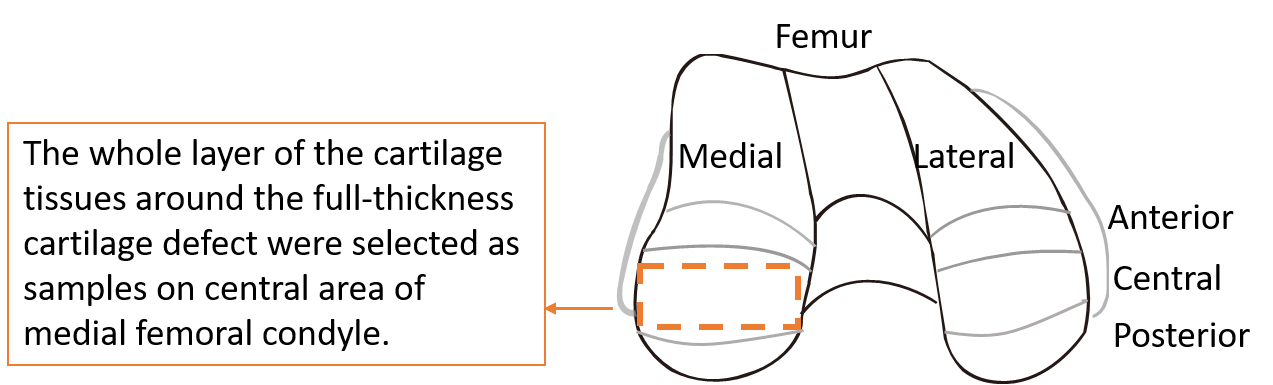

Supplement: Supplementary file 12 — Supplementary Figure 7 [file 41413_2020_109_MOESM12_ESM.tif]

a

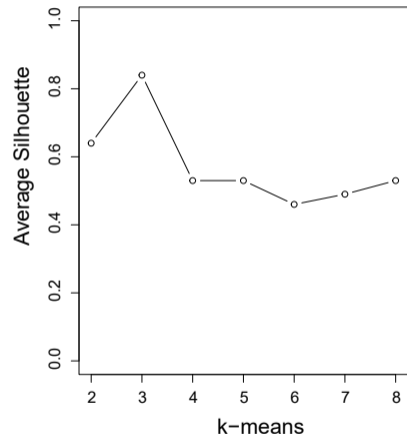

b

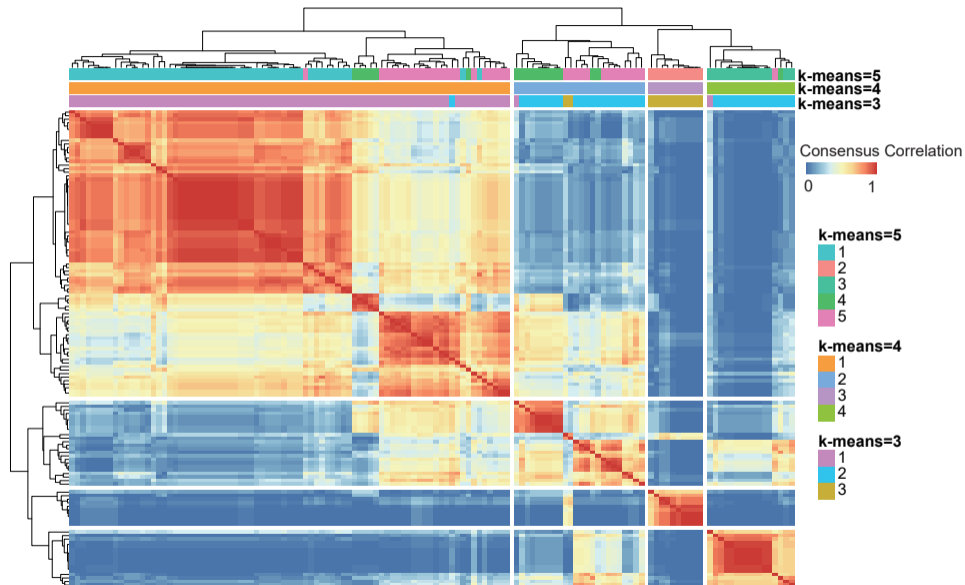

Supplement: Supplementary file 13 — Supplementary Figure 8 [file 41413_2020_109_MOESM13_ESM.pdf]
