# Supplementary material for: Classification of four distinct osteoarthritis subtypes with a knee joint tissue transcriptome atlas
Source: Bone Res. 2020 Nov 12;8:38. doi: 10.1038/s41413-020-00109-x (PMC7658991; doi:10.1038/s41413-020-00109-x)
Supplement: Supplementary file 4 — Supplementary Figure 2 [file 41413_2020_109_MOESM4_ESM.pdf]

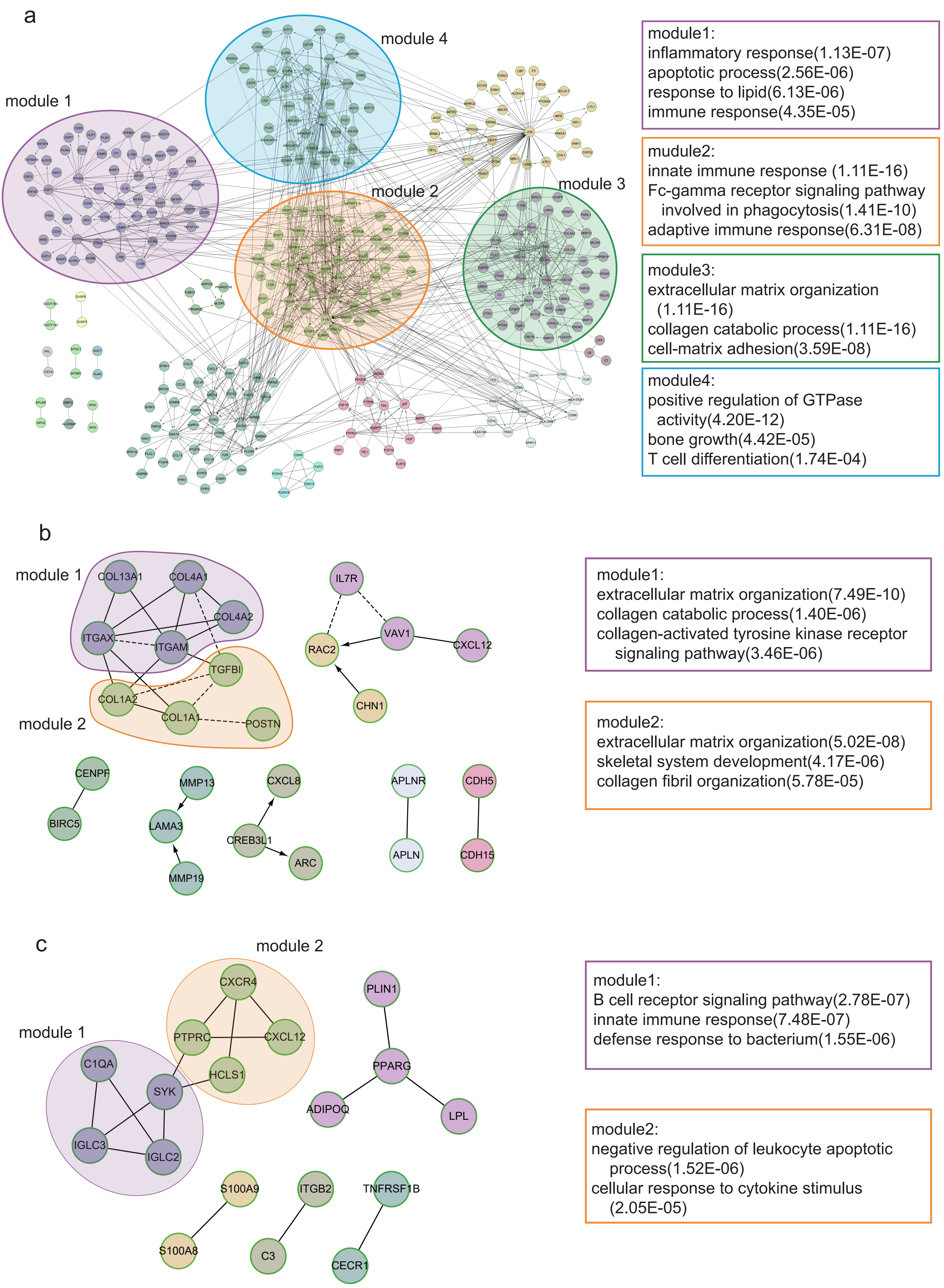
a
module 1
module 4
module 2
module 3
module1:
inflammatory response(1.13E-07)
apoptotic process(2.56E-06)
response to lipid(6.13E-06)
immune response(4.35E-05)
mudule2:
innate immune response (1.11E-16)
Fc-gamma receptor signaling pathway involved in phagocytosis(1.41E-10)
adaptive immune response(6.31E-08)
module3:
extracellular matrix organization (1.11E-16)
collagen catabolic process(1.11E-16)
cell-matrix adhesion(3.59E-08)
module4:
positive regulation of GTPase activity(4.20E-12)
bone growth(4.42E-05)
T cell differentiation(1.74E-04)
b
module 1
module 2
COL13A1
COL4A1
COL4A2
ITGAX
ITGAM
TGFBI
COL1A2
COL1A1
POSTN
IL7R
VAV1
RAC2
CXCL12
CHN1
CENPF
BIRC5
MMP13
LAMA3
MMP19
CXCL8
CREB3L1
ARC
APLNR
APLN
CDH5
CDH15
module1:
extracellular matrix organization(7.49E-10)
collagen catabolic process(1.40E-06)
collagen-activated tyrosine kinase receptor signaling pathway(3.46E-06)
module2:
extracellular matrix organization(5.02E-08)
skeletal system development(4.17E-06)
collagen fibril organization(5.78E-05)
c
module 2
module 1
CXCR4
PTPRC
CXCL12
HCLS1
C1QA
SYK
IGLC3
IGLC2
PLIN1
PPARG
ADIPOQ
LPL
S100A9
S100A8
ITGB2
C3
TNFRSF1B
CECR1
module1:
B cell receptor signaling pathway(2.78E-07)
innate immune response(7.48E-07)
defense response to bacterium(1.55E-06)
module2:
negative regulation of leukocyte apoptotic process(1.52E-06)
cellular response to cytokine stimulus (2.05E-05)
