# Supplementary material for: Classification of four distinct osteoarthritis subtypes with a knee joint tissue transcriptome atlas
Source: Bone Res. 2020 Nov 12;8:38. doi: 10.1038/s41413-020-00109-x (PMC7658991; doi:10.1038/s41413-020-00109-x)
Supplement: Supplementary file 7 — Supplementary Figure 4 [file 41413_2020_109_MOESM7_ESM.pdf]

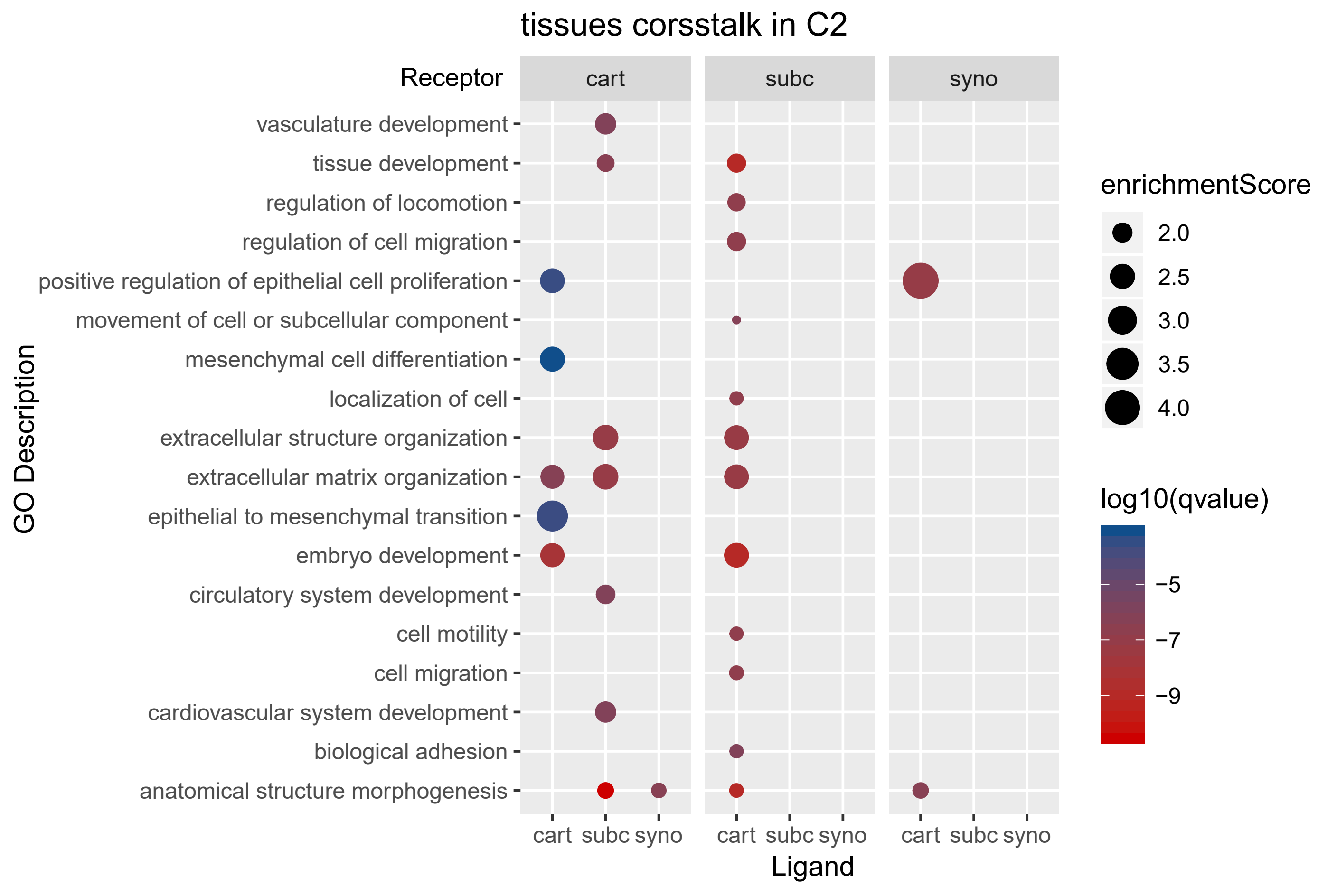
tissues corsstalk in C2
Receptor
cart
subc
syno
vasculature development
tissue development
regulation of locomotion
regulation of cell migration
positive regulation of epithelial cell proliferation
movement of cell or subcellular component
mesenchymal cell differentiation
localization of cell
extracellular structure organization
extracellular matrix organization
epithelial to mesenchymal transition
embryo development
circulatory system development
cell motility
cell migration
cardiovascular system development
biological adhesion
anatomical structure morphogenesis
GO Description
cart subc syno
cart subc syno
cart subc syno
Ligand
enrichmentScore
2.0
2.5
3.0
3.5
4.0
log10(qvalue)
−5
−7
−9
