# Supplementary material for: Classification of four distinct osteoarthritis subtypes with a knee joint tissue transcriptome atlas
Source: Bone Res. 2020 Nov 12;8:38. doi: 10.1038/s41413-020-00109-x (PMC7658991; doi:10.1038/s41413-020-00109-x)
Supplement: Supplementary file 9 — Supplementary Figure 5 [file 41413_2020_109_MOESM9_ESM.pdf]

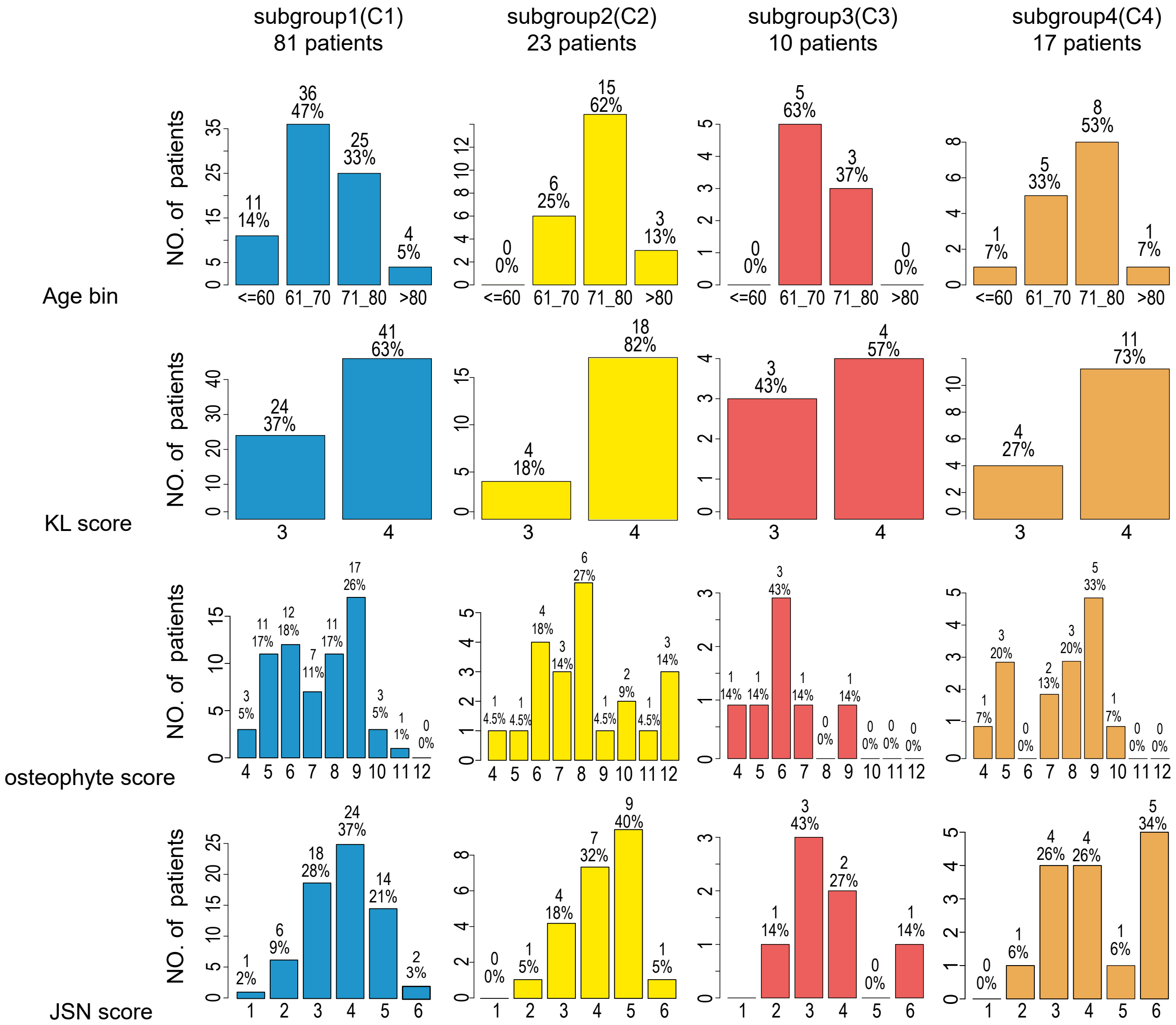

subgroup1(C1)
81 patients
subgroup2(C2)
23 patients
subgroup3(C3)
10 patients
subgroup4(C4)
17 patients
Age bin
KL score
osteophyte score
JSN score
NO. of patients
<=60
61_70
71_80
>80
11 14%
36 47%
25 33%
4 5%
0 0%
6 25%
15 62%
3 13%
0 0%
5 63%
3 37%
0 0%
1 7%
5 33%
8 53%
1 7%
24 37%
41 63%
4 18%
18 82%
3 43%
4 57%
4 27%
11 73%
3 5%
11 17%
12 18%
7 11%
11 17%
17 26%
3 5%
1 1%
0 0%
1 4.5%
1 4.5%
4 18%
3 14%
6 27%
1 4.5%
2 9%
1 4.5%
3 14%
1 14%
1 14%
3 43%
1 14%
0 0%
1 14%
0 0%
0 0%
0 0%
1 7%
3 20%
0 0%
2 13%
3 20%
5 33%
1 7%
0 0%
0 0%
1 2%
6 9%
18 28%
24 37%
14 21%
2 3%
0 0%
1 5%
4 18%
7 32%
9 40%
1 5%
1 14%
3 43%
2 27%
0 0%
1 14%
0 0%
1 6%
4 26%
4 26%
1 6%
5 34%
